# Active Loading of Pectin Hydrogels for Targeted Drug Delivery

**DOI:** 10.3390/polym15010092

**Published:** 2022-12-26

**Authors:** Oraya Vesvoranan, Betty S. Liu, Yifan Zheng, Willi L. Wagner, Joseph Sutlive, Zi Chen, Hassan A. Khalil, Maximilian Ackermann, Steven J. Mentzer

**Affiliations:** 1 Laboratory of Adaptive and Regenerative Biology, Brigham & Women’s Hospital, Harvard Medical School, Boston, MA 02115, USA; 2 Department of Diagnostic and Interventional Radiology, Translational Lung Research Center, University of Heidelberg, 69117 Heidelberg, Germany; 3 Institute of Functional and Clinical Anatomy, University Medical Center of the Johannes Gutenberg-University Mainz, 55131 Mainz, Germany

**Keywords:** pectin, loading, morphometry, microscopy, contact angles

## Abstract

Hydrogels provide a promising method for the targeted delivery of protein drugs. Loading the protein drug into the hydrogel free volume can be challenging due to limited quantities of the drug (e.g., growth factor) and complex physicochemical properties of the hydrogel. Here, we investigated both passive and active loading of the heteropolysaccharide hydrogel pectin. Passive loading of glass phase pectin films was evaluated by contact angles and fractional thickness of the pectin films. Four pectin sources demonstrated mean contact angles of 88° with water and 122° with pleural fluid (*p* < 0.05). Slow kinetics and evaporative losses precluded passive loading. In contrast, active loading of the translucent pectin films was evaluated with the colorimetric tracer methylene blue. Active loading parameters were systematically varied and recorded at 500 points/s. The distribution of the tracer was evaluated by image morphometry. Active loading of the tracer into the pectin films required the optimization of probe velocity, compression force, and contact time. We conclude that active loading using pectin-specific conditions is required for the efficient embedding of low viscosity liquids into pectin hydrogels.

## 1. Introduction

Conventional drug delivery is typically achieved through oral administration [1]. Limited by poor targeting, oral administration often requires high doses and repeated administration. Peptide and protein drugs are particularly inefficient. Protein drugs typically have short circulation times with serum half-lives of only minutes to hours. Enhanced efficiency of drug delivery requires a method of controlling drug availability over time and space, i.e., a method of directing the protein drug to the target organ. Targeted drug delivery offers the possibility of not only minimizing the required dose, but also reducing potentially toxicity [2].

Hydrogels provide a promising method for the targeted delivery of protein drugs. Defined as a two-component system composed of a hydrophilic three-dimensional network capable of retaining a large amount of water [3], hydrogels can often serve as a vehicle without the unintended chemical modification of the polymer or the protein drug. Many hydrogels are capable of preserving drug bioactivity throughout the packaging, storage and delivery process [4]. The ideal hydrogel remains chemically and physically stable prior to targeted delivery, then degrades after drug delivery [5,6].

Hydrogels differ widely in polymer architecture and function [7,8,9]. Hydrogels can theoretically retain the protein drug and growth factors through complicated chemistry involving covalent, electrostatic and hydrophobic interactions [6], but the simplest mechanism is physical encapsulation [10], i.e., the drug is retained in the polymers’ free volume where the relative size of the protein drug and the polymer pores determine the rate of drug release. The most common mechanism of drug release is simple diffusion regulated by the steric interactions between the drug and the polymer network [10,11]. When the mesh size of the network is larger than the drug, the process is dominated by diffusion. Small drug molecules diffuse freely, and diffusion is independent of mesh size. Larger drugs diffuse slower and may require hydrogel erosion for drug release [12].

Loading the protein drug into the hydrogel free volume can be challenging, particularly when there are limited quantities of a drug (e.g., growth factor) and complex physicochemical properties of the hydrogel. In general, protein drugs are embedded during gelation. The protein drug can be mixed with the hydrogel during the gelation process (loading). Alternatively, the protein can be added to preformed gel (post-loading). Typically, post-loading occurs by incubating the liquid protein with the preformed gel. More recently, liquid protein has been incorporated into a liquid injectate. The protein is incorporated in the hydrogel as it jellifies at the site of administration in vivo (in situ loading).

A particularly appealing vehicle for targeted drug delivery is the plant-derived heteropolysaccharide biopolymer called pectin. Pectin has a high content of partially esterified linear chains of (1,4)-alpha-d-galacturonic acid residues and a substantial free volume [13]. Importantly, the entanglement of pectin chains with the surface glycocalyx of mammalian cells [14] creates strong adhesion to visceral organ surfaces [15,16,17,18]. Pectin is bioabsorbable, biodegradable, and cytocompatible. In post-WWII United States, pectin was used as an intravenous volume expander because of the paucity of blood products. To quote one study, “altogether, in patients who had received 4000 cc of pectin solution [intravenously], no changes were encountered which possibly could be attributed to the administration of pectin” [19]. Pectin was evaluated and cleared toxicologically by the Joint FAO/WHO Expert Committee on Food Additives (JECFA) in 1981 [20]. In the United States, pectin is affirmed GRAS (Generally Recognized as Safe), as defined in the Code of Federal Regulations 184.1588 [21]. Pectin is bioabsorbable and widely recognized as a harmless food additive in Europe and North America. Notably, pectin is a common vehicle for “gel-caps” [22].

In this report, we examined the physical parameters involved in the post-loading of the pectin hydrogel. The passive loading of pectin films was assessed by contact angle measurements. Enabled by the translucent properties of pectin films, active loading was assessed using a methylene blue tracer and image morphometry. Optimal post-loading conditions involved a tradeoff between probe velocity, contact time, and compression force.

## 2. Materials and Methods

**Pectin**. The lemon pectin used in this study was obtained from a commercial source (Cargill, Minneapolis, MN, USA). The soy, potato, and sugarbeet pectins were obtained from Megazyme (Bray, Ireland). The proportion of galacturonic acid residues in the methyl ester form determined the degree of methoxylation. High-methoxyl pectins (HMP) were defined as those pectin polymers with a greater than 50% degree of methoxylation. The pectin powder was stored in low humidity at 25 °C.

**Pectin films**. The pectin powder (3% *w*/*w*) was dissolved at 25 °C by a step-wise increase in added water to avoid undissolved powder [23]. Exogenous heat was not used. The complete dissolution of the pectin was achieved using a high-shear 10,000 rpm rotor-stator mixer (L5M-A, Silverson, East Longmeadow, MA, USA). The dissolved pectin was poured into a standard mold for further studies. The pectin films were allowed to equilibrate to ambient 40% relative humidity. 

**Contact angle**. Contact angle measurements were obtained from a sessile 5 uL drop placed at 3 representative areas of the pectin film. The droplets were imaged with a custom Nikon SMZ stereomicroscope system oriented orthogonal to the plane of the film. An image series was acquired using MetaMorph 7.10 software (Molecular Devices, Downingtown, PA USA) time-base corrected images. An image stack, obtained over the observation interval, was digitally combined and the contact angle measured using MetaMorph software.

**Pleural fluid**. Informed consent was obtained from all patients for the anonymized use of discarded tissue and fluid as previously described [24]. Pleural effusion fluid was without clinical or cytologic evidence of inflammation. Fluid was obtained from standard Pleur-Evac drain systems (Teleflex, Morrisville, NC, USA). Patient pleural fluid (N = 10 patients) was pooled to minimize variability. 

**Active loading**. After calibration of the 5 kg load cell (TA-XT plus; Stable Micro Systems, Godalming, Surrey, UK), a 25-mm diameter acrylic disc was mounted to the crosshead over the center of a 25-mm pectin polymer attached to the fixture with proprietary double adhesive (3 M Research Division, St. Paul, MN USA). The loading probe descended at a selectable velocity, compression force, and contact time. Data were acquired at 500 points per second.

**Figure 1:**
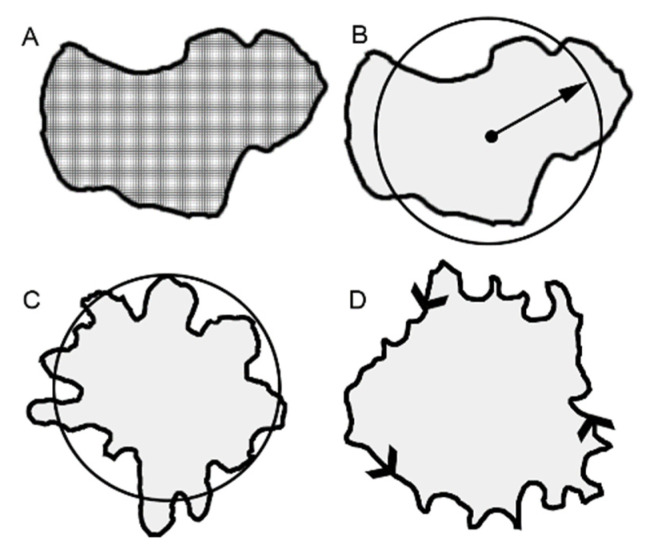
Distinctive morphometric measures of the loaded methylene blue tracer. The total area of the tracer was reflected in several measures that assess cumulative pixel area. Examples of total area (**A**), mean radius (**B**), shape factor (**C**) and perimeter (**D**) are shown.

**Morphometry analysis**. Standard lighting and acquisition settings were used to obtain 12-megapixel images of the tracer patterns. Background subtraction and standard MetaMorph 7.10 software (Molecular Devices) filters were applied. The 14-bit grayscale images were thresholded and distance calibration was performed. The images were measured using MetaMorph’s *Integrated Morphometry* application. *Total area* was the total area of the number of pixels in the image irrespective of intensity variation (Figure 1A). *Total intensity* reflected the sum of the grayscale intensity (pixel brightness) values. *Mean radius* was the measure that reflected the equivalent average radial distance of the object (Figure 1B). *Radial dispersion* was an intensity-weighted “equivalent radius” of a disk whose total integrated intensity was equal to the total integrated intensity of the object. *Shape factor* was the measure that reflected $SF=\frac{4\pi A}{P^{2}}$, where *P* = perimeter and *A* = area. A shape factor value of 1.0 reflected a perfect circle (Figure 1C). *Optical density* (O.D.) was the inverse logarithm of the grayscale transmittance of each pixel expressed as a value of the maximum value. *Integrated O.D*. was the sum of the optical densities of all pixels that made up the object. *Perimeter* was the distance around the border of the region (Figure 1D).

**Statistical analysis.** The statistical analysis was based on measurements in at least three different samples. The unpaired Student’s t test for samples of unequal variances was used to calculate statistical significance. The data are expressed as mean ± one standard deviation. The significance level for the sample distribution was defined as *p* < 0.01. The morphometry curves were best fit curves based on aggregated data from all 3 conditions (contact time, velocity, and compression force); typically, more than 36 replicants per graph.

## 3. Results

**Figure 2:**
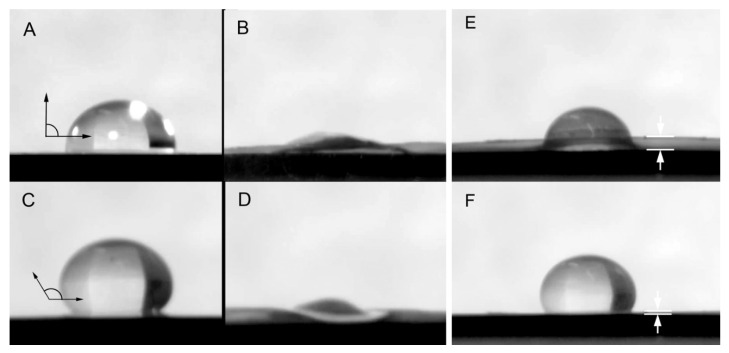
Measurement of contact angle and fractional thickness variation. (**A**) The water contact angle with progressive evaporation and absorption (**B**). (**C**) Pleural fluid contact angle with progressive evaporation and absorption (**D**). Associated with water (**E**) or pleural fluid (**F**) absorption was an increase in fractional thickness of the pectin film (white arrows).

**Figure 3:**
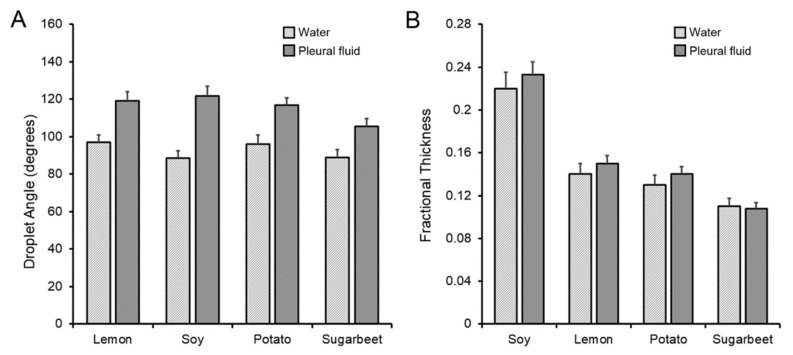
Passive absorption of water and pleural fluid on different plant sources of pectin. Lemon, soy, potato and sugarbeet pectin were compared for droplet contact angle (**A**) and fractional thickness (**B**). The pleural fluid consistently demonstrated larger contact angles than water (*p* < 0.01). There was no difference in fractional thickness (*p* > 0.05) (error bars = 1 SD).

**Figure 4:**
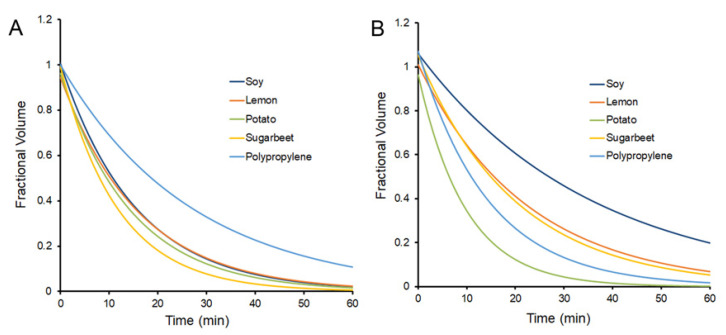
The kinetics of the passive absorption and evaporation of the water (**A**) and pleural fluid (**B**) droplets was assessed by timelapse video and planar morphometry (MetaMorph, Molecular Devices). The fractional residual volume was studied in all 4 plant sources as well as a polypropylene evaporative control. The median curve of 5 replicate samples is shown.

**Passive post-loading of glass phase pectin films**. The contact angle provided insights into the physicochemical interaction at the hydrogel interface (Figure 2). Different plant sources of the pectin hydrogel were compared to water and human pleural fluid, a biologic fluid containing 1.5 gm/dL of protein [25]. All four variant pectins demonstrated contact angles of 88° with water and 122° with pleural fluid (*p* < 0.05) (Figure 3A). Fractional thickness, reflecting initial interface swelling of the film, was similar (*p* > 0.05) (Figure 3B). The rate of water absorption into the film was more rapid with water than pleural fluid (Figure 4). Of note, polypropylene provided the evaporative nonabsorbent surface control.

**Figure 5:**
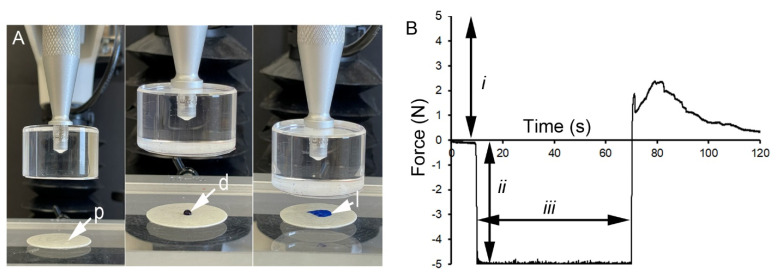
Active loading of the pectin films. (**A**) The pectin film (p) is attached to the fixture surface with a double-sided adhesive. A 50 uL water droplet (d) containing the methylene blue tracer was placed on the pectin surface. The droplet was loaded (l) into the pectin film with an inert acrylic probe with an interface parallel to the pectin surface. (**B**) The selectable features of the loading process included the velocity of the probe (*i*), the compressive force of the probe (*ii*) and the contact time (*iii*) of probe compression. In this example, the probe velocity was 0.25 mm/s, the compression force was 5 N and the contact time was 60 s.

**Active post-loading.** The inefficiency of passive drug loading suggested the potential utility of active compression of the drug into the pectin film (Figure 5A). Because pectin films are translucent, we used the colorimetric tracer methylene blue to evaluate the efficiency of droplet loading into the glass phase pectin films. The threshold of methylene blue tracer detection was 0.00625 mg/mL with a saturation plateau of 0.5 mg/mL. Loading parameters of probe velocity, compression force, and contact time were recorded at 500 pps (Figure 5B). The embedded tracer was imaged and analyzed by quantitative morphometry.

**Figure 6:**
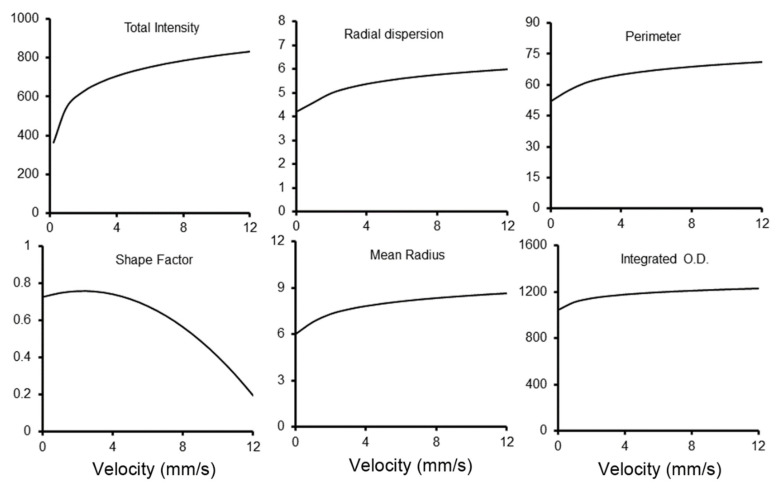
Morphometric analysis of methylene blue loading with variable load velocity. The morphometry measures reflecting the area or radial dispersion of the tracer increased toward an apparent velocity plateau of 5 mm/s. Shape factor demonstrated progressive irregularities with increasing velocity. The curves reflect the aggregate best fit curve based on replicate measures of contact time and compressive force.

**Probe velocity.** Probe velocity was assessed because of its potential impact on droplet spreading at the probe-pectin interface. We varied the probe velocities from 0.25 mm/s to 10 mm/s with distance resolution < 0.002 mm (Figure 6). At probe velocities less than 2 mm/s, there was limited lateral distribution of the tracer on the pectin interface. With increasing velocity, the total area and radial dispersion increased, but with increasingly irregular boundaries. At 10 mm/s, probe velocity was associated with shape factors below 0.5, suggesting Hele-Shaw flows (Figure 6).

**Figure 7:**
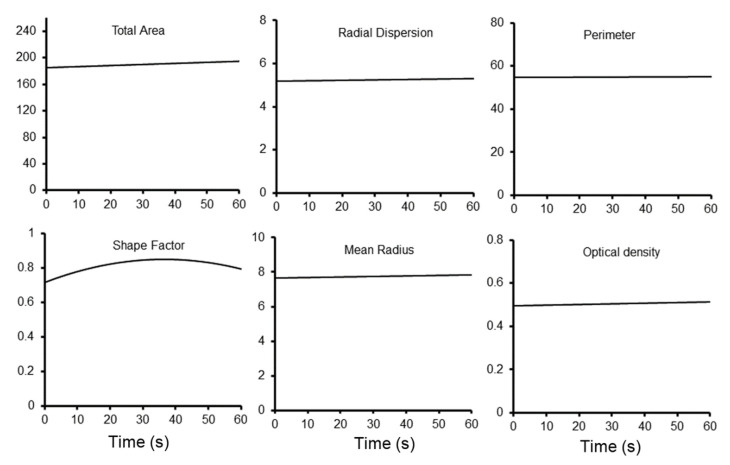
Morphometric analysis of methylene blue loading with variable contact time. The morphometry measures did not demonstrate any dependence on contact time. A minimum of 15 s appeared to be required to insure adequate wetting. The curves reflect the aggregate best fit curve based on replicate measures of probe velocity and compressive force.

**Contact time**. The length of time the loading probe was in contact with the pectin was also varied. The probe contact time was varied from 2 s to 60 s (Figure 7). There was limited impact of contact time on tracer dispersion except for one unexpected observation. If the contact time was less than 15 s, we could routinely detect residual tracer on the acrylic loading probe. The minimum effective contact time for the methylene blue tracer was 15 s.

**Figure 8:**
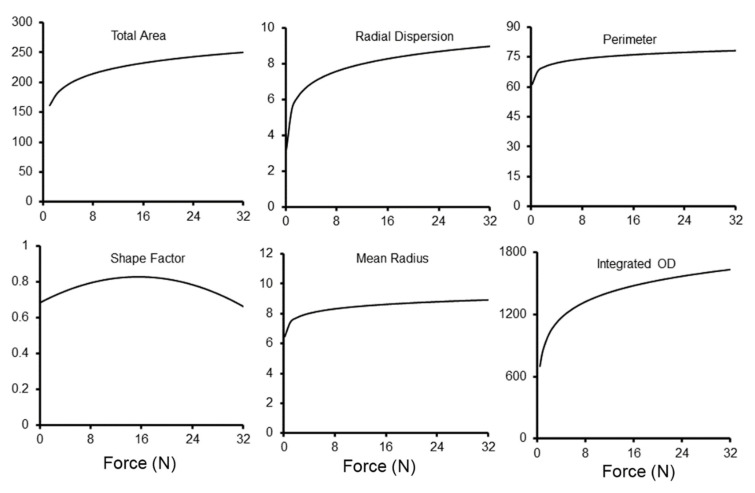
Morphometric analysis of methylene blue loading with variable compression force. The morphometry measures reflecting the area or radial dispersion of the tracer increased toward an apparent plateau of 10 N. The morphometric measures continued to increase marginally with added force. The curves reflect the aggregate best fit curve based on replicate measures of contact time and velocity.

**Compression force**. The force used to compress the droplet had a significant impact on the methylene blue loading (Figure 8). The probe compression force was varied from 0.25 N to 30 N. There was a steep increase in tracer dispersion to 10 N of compression. There was diminishing detectable impact on methylene blue embedding with compression force greater than 10 N. Doubling film thickness had no effect on tracer dispersion.

## 4. Discussion

In this report, we identified several empirical considerations for the active loading of pectin films. (1) Surface energies limited passive loading of both proteinaceous and nonproteinaceous liquids into the pectin film. Although contact angles varied between different sources of pectin, droplet evaporation produced practical limits to passive loading. (2) Interfacial flow produced by active loading increased dispersion but became more unpredictable at higher probe velocities. Probe velocities greater than 5 mm/s produced irregularities characterized by variable fingering at the margin of the detectable tracer. (3) Compression produced the most efficient loading of the tracer into the pectin matrix. Consistent with Darcy’s law, the pressure gradient created by the probe compression force produced a linear increase in loading efficiency with a plateau near 5 N. We conclude that active loading of low viscosity liquids into pectin hydrogels can be efficiently performed with probe velocity less than 5 mm/s, and compression force greater than 5 N.

The initial interaction of the low viscosity liquid and the pectin surface is determined by surface forces. Contact angles were a useful measure of the surface tension at the liquid-pectin interface. Contact angles not only evaluated the interaction energy between the pectin and liquid droplet [26,27], but also provided practical information about the physicochemical properties of the pectin matrix [28]. Here, we investigated the surface properties of four pectin variants. Although there was some variability, none of the pectin sources were associated with rapid spreading or absorption. These findings suggested that an active process would be required for efficient drug loading.

In our process of active loading, the probe movement created interesting dynamics. As the probe descended, the increasingly small gap between the two parallel surfaces accelerated the droplet fluid in directions perpendicular to the interface. At higher probe velocities, we detected fingering irregularities at the margin of the loaded tracer. The fingering of the tracer was reminiscent of Hele-Shaw flows [29,30,31]. Moreover, also consistent with microflow instabilities, a significant decrease in shape factor (more irregular margins) was seen at probe velocities greater than 5 mm/s. 

The most significant impact on drug loading was probe compression of the droplet on the pectin film. We observed a linear increase in tracer loading with an increase in compression force to 5 N and an apparent plateau above 5 N. Consistent with the Darcy relationship defined for hydraulic matrices [32], the increased pressure gradient created by probe compression increased flow into the porous pectin matrix. Parenthetically, we note that the intrinsic permeability value (k) of the Darcy relationship potentially reflects the hydration state of the pectin films.

As a surrogate for testing the contact angle of protein drugs, we used pleural fluid obtained from human patients. Pleural fluid is a low viscosity biologic fluid with a high concentration of charged proteins. The mean protein concentration of pleural fluid is 1.5 gm/dL with approximately 1 gm of the negatively charged albumin [33]. Pleural fluid was of particular interest because of its special relationship to pectin. Pleural fluid is the lubricant for the pleural glycocalyx [34]. Importantly, the pleural glycocalyx has been shown to have a remarkable structural similarity to pectin [35]. Our studies showed an increased contact angle compared to water. We speculate that these surface properties may reflect the fluid’s physiologic role in the maintenance and lubrication of the pleural surface.

Methylthioninium chloride [36], commonly called methylene blue, is a blue salt used in a variety of clinical applications, including as a tissue stain [37], intravital dye [38], lymphatic tracer [39], and polypectomy marker [40]. We used methylene blue in these experiments because it was apparently nonreactive and it provided excellent contrast for imaging. As a tracer, methylene blue demonstrated a linear dose–detection relationship to 0.5 mg/mL and a colorimetric plateau above 0.5 mg/mL. Because of this saturation plateau, quantitative assessment of tracer loading was limited to morphometric pattern analysis. 

Finally, our studies highlight the multi-functional potential of the pectin biopolymer. In previous work, we have shown that pectin films strongly bind to the surface glycocalyx of visceral organs with a mechanism of branched chain entanglement [14,41,42]. The strong adhesivity has led to a proposed function of pectin films as a sealant for visceral organ injuries. Pectin has demonstrated the ability to seal pleural air leaks in the lung [17,43] and buttress bowel serosa [44]. Here, we show that pectin free volume can also contribute to drug delivery. As a surrogate for both proteinaceous and nonproteinaceous drugs, the tracer was effectively loaded into glass phase pectin films. The implication of these findings is that drugs can now be delivered directly to the surface of injured organs. We anticipate that pectin films will be a useful delivery mechanism in a range of potential the treatments, including growth factors, antibiotics, anti-inflammatories, and coagulation factors. The process of active loading will facilitate these studies and likely broaden the potential applications for targeted drug delivery.

## Data Availability

The data presented in this study are available upon request.

## Abbreviations

GRAS, Generally Recognized as Safe; HMP; high-methoxyl pectin; JECFA, Joint FAO/WHO Expert Committee on Food Additives; OD; optical density.
